# A Novel In Vivo Method Using *Caenorhabditis elegans* to Evaluate α-Glucosidase Inhibition by Natural Products for Type 2 Diabetes Treatment

**DOI:** 10.3390/ph17121685

**Published:** 2024-12-13

**Authors:** María Pilar de Torre, José Luis Vizmanos, Rita Yolanda Cavero, María Isabel Calvo

**Affiliations:** 1 Department of Pharmaceutical Sciences, School of Pharmacy and Nutrition, University of Navarra, Irunlarrea 1, 31008 Pamplona, Spain; mdetorre@alumni.unav.es; 2 Department of Biochemistry & Genetics, School of Sciences, University of Navarra, Irunlarrea 1, 31008 Pamplona, Spain; jlvizmanos@unav.es; 3 Department of Environmental Biology, School of Sciences, University of Navarra, Irunlarrea 1, 31008 Pamplona, Spain; rcavero@unav.es; 4 IDISNA—Instituto de Investigación Biosanitaria de Navarra, 31008 Pamplona, Spain

**Keywords:** *C. elegans*, hyperglycemia, *O. vulgare*, in vitro digestion

## Abstract

Background: Non-insulin-dependent diabetes mellitus, or type 2 diabetes, is one of the diseases of greatest concern worldwide, and research into natural compounds that are capable of regulating glycemia and insulin resistance is therefore gaining importance. In the preclinical stages, *Caenorhabditis elegans* is considered a promising in vivo model for research into this disease. Most studies have been carried out using *daf-2* mutant strains and observing changes in their phenotype rather than directly measuring the effects within the worms. Methods: We evaluated the in vitro α-glucosidase inhibition of two oral formulations of *Origanum vulgare* before and after a simulated gastrointestinal digestion process. After confirming this activity, we developed a method to measure α-glucosidase inhibition in vivo in the *C. elegans* wild-type strain. Results: The crude extract showed a similar IC50 value to that of acarbose (positive control), before and after gastrointestinal digestion. Formulation 1 also showed no differences with the positive control after digestion (111.86 ± 1.26 vs. 110.10 ± 1.00 µL/mL; *p* = 0.282). However, formulation 2 showed a higher hypoglycemic activity (59.55 ± 0.85 µL/mL; *p* < 0.05). The IC50 values obtained in the in vivo assays showed results that correlated well with the in vitro results, so the proposed new method of direct quantification of the in vivo activity seems suitable for directly quantifying the effects of this inhibition without the need to measure changes in the phenotype. Conclusion: A novel, simple and reliable method has been developed to directly determine pharmacological activities in an in vivo model of wild-type *C. elegans*. This allows the hypoglycemic activity to be directly attributed to in vivo treatment without quantifying phenotypic changes in mutant strains that may arise from other effects, opening the door to a simple analysis of in vivo pharmacological activities.

## 1. Introduction

After ingestion, sugars and carbohydrates are digested and reduced to glucose, which is absorbed in the intestine. The increase in blood sugar promotes the release of insulin in the pancreas so that glucose can enter the cells as a cellular nutrient. In addition, it also promotes the formation of glycogen in the liver. All of this reduces blood sugar and keeps it at levels that are considered normal. The pancreas also produces glucagon, which stimulates the breakdown of glycogen when glucose is needed and there is a lack of intake of it. This is all part of an endocrine feedback system that controls blood sugar and the body’s energy balance. However, this balance can be altered, leading to diabetes or increased blood glucose levels, which can lead to multiple complications. There are three main types of diabetes, depending on the origin of the problem [1].

The first type of diabetes (type 1 diabetes, T1D) affects 5–10% of diabetic patients and is autoimmune, as the body itself destroys the β cells of the pancreas [2]. For its treatment, patients need to inject insulin daily, since this function of the pancreas is partially or completely disabled.

Non-insulin-dependent diabetes mellitus (NIDDM) or type 2 diabetes (T2D) is the most common form of diabetes and affects 90–95% of patients. Its increasing prevalence makes it a disease of global concern [2]. In this case, there is no adequate response to insulin (insulin resistance). This means that glucose cannot enter the cells for storage as an energy source and accumulates in the blood. This type of diabetes develops slowly over time and appears more frequently in overweight people. In addition, it can occur silently and asymptomatically. Once detected, it is important to control blood sugar levels to keep the resistance under control with changes in diet and lifestyle. Its treatment begins with daily oral medications that act on different pathways (DPP-4, GLUT-1, α-glycosidase, etc.). If blood sugar levels are kept under control, monotherapy is sufficient.

The third type of diabetes, gestational diabetes, can occur during pregnancy. Both mother and child may develop diabetes if blood sugar levels are not controlled during pregnancy.

In all three types of diabetes, it is important to educate the patient and select the optimal treatment. When the disease is not treated effectively, high blood sugar levels can cause tissue damage due to overproduction of superoxide in the cells and lead to serious complications such as retinopathies, limb amputations, neuropathies, kidney and cardiovascular diseases or even premature death.

Most of the research into the development of treatments has focused on T1D, given the severity of its complications and the absence of prevention strategies due to its autoimmune nature. However, in the case of T2D, up to 530 million adults [3] and an increasing number of children [2] worldwide could be affected by this pathology, so the development of natural alternatives for its prevention and treatment could be very useful.

One of the most commonly used drugs for T2D is acarbose, which inhibits the α-glucosidase pathway and is effective in delaying the intestinal absorption of polysaccharides and disaccharides. This enzyme, located in the membranous epithelium of the small intestine, is responsible for the breakdown of glucose from ingested disaccharides. Acarbose has some relatively common digestive side effects (flatulence, stomach pain and diarrhea) [4], as well as rarer ones such as nausea, vomiting, increased liver enzymes and indigestion [5].

Research into natural compounds that are capable of regulating blood sugar and insulin resistance is becoming increasingly important, either as an alternative treatment or as an adjuvant to existing treatments. In this regard, it has been shown that there are medicinal plants with α-glucosidase inhibitory activity that generate fewer side effects than acarbose [6,7,8,9,10]. More than 400 traditional medicinal plants are known to have antidiabetic properties. However, only a few of them have been sufficiently studied scientifically to demonstrate their effectiveness [11,12,13,14,15,16].

*Caenorhabditis elegans* is a good in vivo model for the study of antidiabetic bioactivity [17]. It was the first multicellular organism with a complete genome sequence [18] and is widely used in biomedical research due to its homology with various basic human processes [19,20,21], its low cost and its short life cycle.

Most studies on insulin resistance and antidiabetic activity with this model have been carried out using *daf-2* (an insulin-like receptor gene) mutant strains. The effects of the tested compounds are generally attributed to the appearance of physiological changes in the worm [17,22,23,24], but without measuring the pharmacological activity inside it. In this context, it seems necessary to develop a simple and reliable method in this model that allows for the direct quantification of the pharmacological activity of these compounds.

In a previous study, we demonstrated that a hydroalcoholic extract of *Origanum vulgare* exhibited significant antioxidant activity both in vitro and in vivo [25]. However, according to the literature, this medicinal plant has multiple different pharmacological activities that could be useful in age-related metabolic pathologies, such as T2D [26,27,28]. Therefore, we set out to develop an analysis method that would be able to easily quantify hypoglycemic activity in an in vivo model, directly and independently of its possible physiological effects. To this end, we began by evaluating the hypoglycemic activity of two oral formulations with extracts of *Origanum vulgare* L. subsp *vulgare* in vitro, before and after a simulated gastrointestinal digestion process. After confirming the in vitro activity, the hypoglycemic activity was tested in vivo using the newly developed method. The results obtained show a correlation between the in vitro and in vivo assays and the robustness of the method.

## 2. Results and Discussion

### 2.1. In Vitro α-Glucosidase Activity Assay

**Figure 1:**
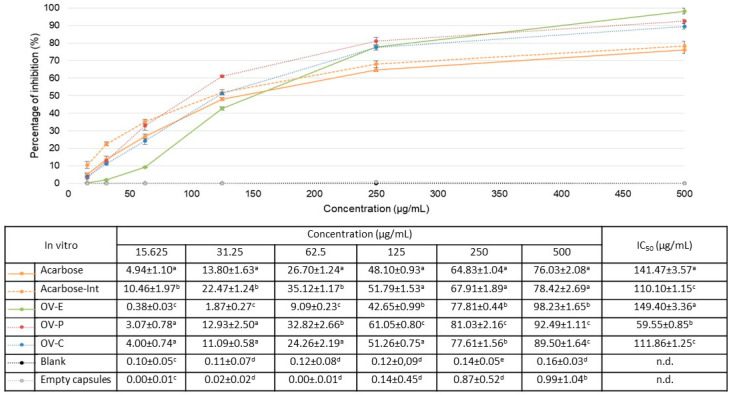
In vitro α-glucosidase inhibitory activity, expressed as the percentage of inhibition (mean ± SD µg/mL). The orange line represents the data obtained with the positive control before (Acarbose—solid line) and after (Acarbose Int.—dashed line) digestion. The green solid line represents the data obtained with the hydroalcoholic extract before digestion (OV–E); the red dashed line represents the data obtained with the dry powder extract (formulation 1) after gastrointestinal digestion (OV-P); and the blue dashed line represents the data with capsules (formulation 2) after gastrointestinal digestion (OV-C). The gray dashed line represents the data from empty capsules, and the black dashed line represents the data from capsules after gastrointestinal digestion. The table below shows the IC_50_ values (mean ± SD µg/mL). In columns with the same concentration, different letters indicate values with significant differences (*p* < 0.05), and the same letters indicate values that do not show significant differences (*p* > 0.05); n.d.: not determined.

First, the inhibitory activity of α-glucosidase in two formulations (powder, formulation 1; and hard gelatin capsules, formulation 2) of a hydroalcoholic extract of *O. vulgare* L. subsp. *vulgare* was tested in vitro. For this purpose, the assay described by Matsui et al. [29] was used, which spectrophotometrically determines the inhibition of this enzyme using a substrate that breaks down into a yellow compound and glucose after exposure to α-glucosidase. Acarbose, one of the drugs that are marketed for the oral treatment of T2D, was used as a positive control, since it competitively inhibits the action of intestinal α-glucosidase. This compound has already been used as a control by Rodríguez-Solana et al. [30] (1 mg/mL), showing an inhibition rate of α-glucosidase of 78.34%. In our assay (Figure 1), acarbose at 0.5 mg/mL showed an inhibition percentage of 76.03 ± 2.08% before being subjected to the digestion process and 78.42 ± 2.69% after this process. The undigested hydroalcoholic extract of *O. vulgare* (OV-E extract) showed a similar in vitro effect to that of acarbose, with IC_50_ values of 149.40 ± 3.36 and 141.47 ± 3.57 µg/mL, respectively (*p* > 0.05).

Methanolic extracts of *O. vulgare* showed an α-glucosidase inhibition percentage of 58.41% at a dose of 10 mg/mL [8]. Previous studies with aqueous and ethanolic extracts at a concentration 10 times lower (1 mg/mL) demonstrated that the extraction solvent is directly related to this hypoglycemic activity (94% vs. 80% inhibition) [31]. *Origanum vulgare* is rich in phenolic compounds, flavonoids and phenolic acids, such as rosmarinic acid, apigenin, luteolin, quercetin and scutellarein [32], and several studies relate these compounds to antidiabetic activity through various mechanisms of action [33].

However, studies on the bioaccessibility and/or bioavailability of these compounds during gastrointestinal digestion processes are very scarce and show contradictory results due to the use of different extraction solvents and tested doses [34,35]. Nevertheless, all of them agree that the bioaccessibility and/or bioavailability of these compounds is low and recommend that further research be conducted considering the inclusion of the extract in encapsulation systems or other types of pharmaceutical formulations.

Overall, the data showed that enzyme inhibition was increased in both formulations after the simulated intestinal digestion process, where this enzymatic activity takes place. This increase in activity was not related to excipients or the digestion enzymes, since both negative controls did not show inhibitory activity (the highest being 0.16 ± 0.03%; *p* < 0.05—Figure 1). On the one hand, the encapsulated extract (111.86 ± 1.25 µg/mL) did not show statistical differences with the intestinal fraction of the positive control (110.10 ± 1.15 µg/mL; *p* = 0.282), so its action was similar to that of acarbose. However, the powdered extract showed the greatest hypoglycemic effect, since the IC_50_ value was the lowest (59.55 ± 0.85 µg/mL; *p* < 0.05).

A previous study with flavonoid fisetin obtained from the leaves of *Rhus succedanea* L. [36] also showed a higher α-glucosidase inhibitory activity than that of acarbose, and its encapsulation in nanoparticles did not improve the inhibitory activity either. Using a computational molecular docking approach, the authors explained their results in relation to the structural determinants that are involved in the interaction between flavonoids and the enzyme, considering that the 3′,4′-dihydroxyl groups of the B ring in flavonoids are crucial to achieve direct binding to the active site residues. In our case, the flavonoid content was higher in the intestinal fraction of the capsule (OV-C) than in the intestinal fraction of the powder (OV-P). Thus, the observed effect could be due to the fact that in the powder, the flavonoid binding site was cleared and released from the chemical groups mentioned above, while in the case of the capsules, the physical protection that they provide would not facilitate this clearance. In summary, digestion seems to release certain compounds that are important for inhibition, which are more effective in the case of the extract administered in powder form (formulation 1) than in the same extract encapsulated (formulation 2). In this sense, this study demonstrates that the pharmaceutical form influences in hypoglycemic activity after a gastrointestinal digestion process. It has been reported that the bioaccessibility of polyphenols depends on the composition of the matrix [37]. This could explain the differences observed between the two oregano formulations.

### 2.2. In Vivo α-Glucosidase Activity Assay

**Figure 2:**
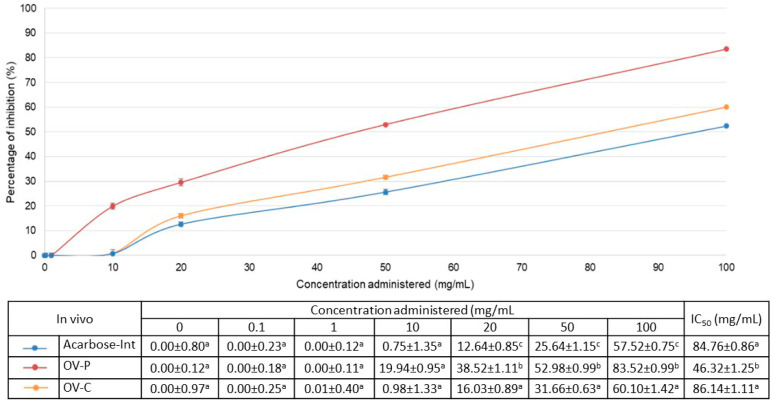
In vivo α-glucosidase inhibitory activity expressed as the percentage of inhibition (mean ± SD%). The red solid line represents the data obtained with the dry powder after gastrointestinal digestion (OV-P); the solid blue line represents the data with the capsules after gastrointestinal digestion (OV–C); the orange solid line represents the data from acarbose after digestion (Acarbose-Int). The table below shows the percentage of inhibition values (mean ± SD%). In columns with the same concentration, different letters indicate values with significant differences (*p* < 0.05), and the same letters indicate values that do not show significant differences (*p* > 0.05).

Although absorbable intestinal fractions have been shown to inhibit this enzymatic activity in vitro, we wondered whether this effect would be maintained in a living organism. To establish this, we determined the activity of these pharmaceutical forms and acarbose in *C. elegans* after simulated digestion. Thus, we designed a simple method that would allow us to directly quantify this activity, rather than through their physiological or behavioral effects on this organism. Our aim was to use this method to confirm that the extract was ingested and as a preliminary in vivo test of its activity that could be correlated with physiological responses obtained in other studies [22,23,24]. Figure 2 presents the results of this analysis in vivo.

There are numerous studies analyzing the hypoglycemic activity of *Oregano vulgare* in cell cultures [38] and in alloxan-induced diabetic rats [39]. In all cases, the results were very promising for the aqueous extract. The *O. vulgare* extract reduced total cholesterol and low-density lipoprotein cholesterol levels and, conversely, increased high-density lipoprotein cholesterol levels. However, this is the first time this activity has been studied in the *C. elegans* nematode for this species, so the results obtained cannot be directly compared. The antioxidant activity of various extracts has been previously investigated by our research group [40].

### 2.3. Correlation of In Vitro Versus In Vivo α-Glucosidase Activity Assay

The results of the in vivo analysis, expressed as the percentage of inhibition, showed that the encapsulated extract (OV-C) had a similar behavior to the positive control (Acarbose-Int) and, again, the extract administered in powder form (OV-P) showed a significantly greater inhibition of enzymatic activity than the positive control at any of the concentrations: 52.98 ± 0.99% versus 31.66 ± 0.63% at 50 mg/mL, respectively (*p* < 0.05). Both results were similar to those obtained with the in vitro assay.

**Figure 3:**
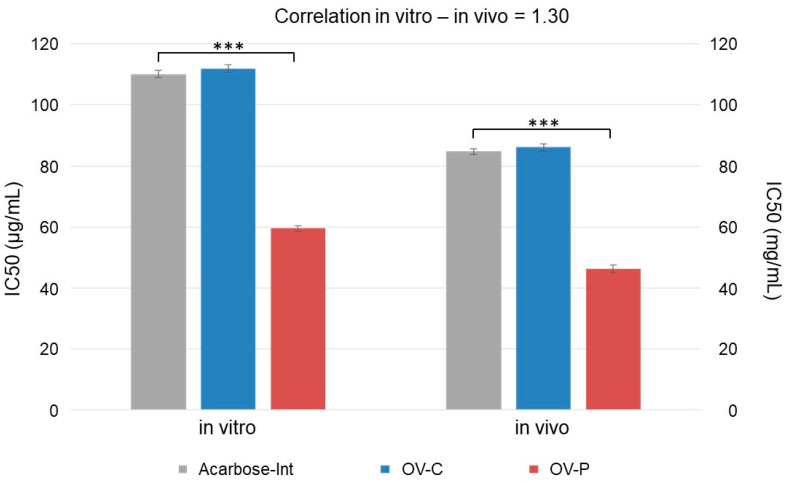
IC_50_ values of intestinal absorbable fractions of acarbose (gray, Acarbose-Int) extract administered in capsules (blue, OV-C) and extract in powder form (red, OV-P). On the left, the values from the in vitro assay are presented (mean ± SD µg/mL). On the right, the values from the in vivo assay are presented (mean ± SD mg/mL). ***: statistical differences, *p* < 0.05.

In vitro assays are used to demonstrate the intrinsic activity of compounds under laboratory conditions, while in vivo assays focus on their potential effects on a living organism, confirming their effects. Sometimes, there is no direct correlation between the results of both types of tests, but in our case, the correlation was 1.30 (Figure 3).

Although the high correlation observed between both types of assays (in vitro and in vivo) needs to be validated in other independent studies, the new in vivo approach proposed in this work provides a novel analysis system to test new treatments for diabetes without having to quantify gene expression or their potential physiological effects (which can be subjective and time-consuming) and also directly attributing the hypoglycemic effect. The proposed assay was replicated three times using independent worm stocks for validation, and in all cases, the results showed robustness and reproducibility, with a coefficient of variation of less than 20% (between 1.87 and 6.72%). Furthermore, as previously indicated, this assay also allows us to confirm that the worms are effectively eating the extract. In summary, it is a direct, simple and novel assay that can be adapted to other investigations of in vitro pharmacological activities that require an in vivo counterpart.

## 3. Materials and Methods

### 3.1. Extract Preparation and Formulations

The *Origanum vulgare* L. subsp. *vulgare* used in this study was collected in Santacara (Navarra, Spain), and, after its authentication by Dr. R.Y. Cavero, a reference specimen (PAMP21629) was deposited in the herbarium of the Department of Environmental Biology (School of Sciences, University of Navarra, Pamplona, Spain).

The two pharmaceutical forms used were prepared as described by de Torre et al. [40]. Briefly, 10 g of air-dried flowering parts were macerated at 4 °C in 250 mL of 50% *v*/*v* ethanol for 24 h and gravity-filtered, repeating the process for a total of four times. The extract (OV-E) was concentrated using a rotary evaporator and lyophilized (DER 3:1, 30% rosmarinic acid). The two simple oral pharmaceutical forms (divided powder and hard gelatin capsules) of the genuine extract were subsequently prepared with 30% of rosmarinic acid and silicon dioxide (E551) as antiaglomerant.

### 3.2. In Vitro Gastrointestinal Digestion Process

The in vitro gastrointestinal digestion process was carried out according to Gayoso et al. [41], with some modifications as previously described by de Torre et al. [40]. Each pharmaceutical form (equivalent to 500 mg of extract) was subjected to the gastrointestinal digestion process, obtaining the intestinal absorbable fraction either from the powder (OV-P), capsules (OV-C) or from the positive control (Acarbose-Int), together with a control with two empty capsules and a blank with the excipients and without plant extract. The obtained fractions were lyophilized (Cryodos50, Telstar, Barcelona, Spain).

### 3.3. In Vitro α-Glucosidase Activity Assay

**Figure 4:**
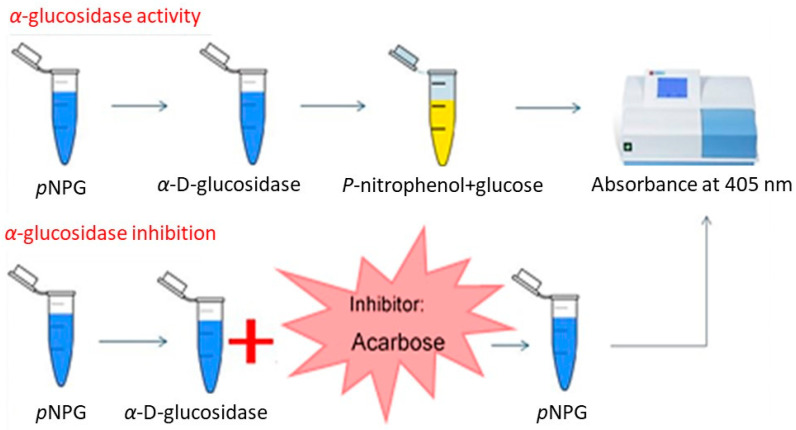
Schematic of α-glucosidase inhibition and activity. P-NPG (4-nitrophenyl-α-D-glucopyranoside) is a disaccharide that is degraded by the enzyme into P-nitrophenol (a compound which turns yellow and is quantifiable at 405 nm) and glucose. In the presence of any α-glucosidase inhibitor (e.g., acarbose), the yellow coloration does not occur due to the absence of enzymatic activity.

The inhibitory activity of α–glucosidase can be quantified using the disaccharide 4-nitrophenyl-α-*D*-glucopyranoside (p-NPG) as a substrate [10,29,42,43]. The enzyme breaks the α-chemical bond of the substrate, releasing the carbohydrate D-glucopyranoside and the phenolic compound 4-nitrophenol. The latter compound turns yellow at a pH of 6.8, allowing for its quantification at 405 nm (Figure 4). The higher the inhibitory activity of the extract is, the less yellow the solution will be. To enhance the color, a basic solution must be added (to reach the optimal pH 6.8) [34] so that there are enough electrons for the 4–nitrophenol to stabilize [35].

The α–glucosidase enzyme was obtained from *Saccharomyces cerevisae* (#G0660, Sigma–Aldrich Co., St. Louis, MO, USA) and was resuspended in 0.1 M PBS buffer, at a pH of 6.8 [44], at a concentration of 0.5 U/L (within the suggested range of 0.2–1 U/L [45]). The substrate used was 4-nitrophenyl-α-*D*-glucopyranoside (#*N*1377, Sigma-Aldrich Co., St. Louis, MO, USA), and after a several titration tests, we found that the optimal concentration was 1 mM in 0.1 M PBS buffer, at a pH of 6.8. Both elements must be kept cold during handling.

The different lyophilized samples were also diluted in 0.1 M PBS buffer, with a pH of 6.8, at six serial concentrations (1000–0.03125 µg/mL). In addition, 120 µL acarbose (#A8980, Sigma-Aldrich Co., St. Louis, MO, USA) was used as a positive control at the same concentrations, and 120 µL of the sample solvent (PBS) was used as a negative control. Finally, 20 µL of PBS was used for blanks instead of the enzyme (2 µL 0.5 U/L in PBS), and the volume of substrate was constant for each condition (20 µL). Samples, acarbose, PBS and enzymes were added to a 96-well plate and incubated for 15 min at 37 °C with shaking. The substrate was then added and incubated under the same conditions for a further 15 min. After that, to stop the reaction and prevent biodegradation, 80 µL of 0.15 M Na_2_CO_3_ was added to each well.

Finally, absorbance was quantified with the Power WaVe XS spectrophotometer (BioTek Instruments, Winooski, VT, USA) at 405 nm. Results were processed with BioTek KC Junior data analysis software. α-Glucosidase inhibition was calculated by using the following equation:

$$Inhibition\;\left(\%\right)=\left(1-\frac{\left(\left(Abs_{S}-Abs_{SB}\right)-\left(Abs_{C}-Abs_{CB}\right)\right)}{Abs_{C}-Abs_{CB}}\right)\times 100$$

where inhibition (%, percentage of inhibition) is obtained as the absorbance of the sample (*Abs_S_*) minus the absorbance of the negative control (*Abs_C_*). The absorbances of their respective blanks (*Abs_SB_*: Sample Blank Absorbance and *Abs_CB_*: Negative Control Blank Absorbance) must be subtracted from both values. The obtained value is relative to the value of the absorbance of the control.

The inhibitory concentration (IC_50_) was calculated using GraphPad Prism v 4.00 analysis software (GraphPad Software, Boston, MA, USA).

### 3.4. In Vivo α-Glucosidase Activity Assay

*C. elegans* N2 *Bristol* was cultured as previously described [25]. All assays were performed in 6-well cell culture plates with 4 mL of Nematode Growth Medium (NGM) per well at 20 °C. Digested samples of each formulation and acarbose as positive control were resuspended in distilled water at different increasing concentrations: 0, 0.1, 1, 10, 20, 50 and 100 mg/mL. A total of 40 µL of each solution was added per well.

**Figure 5:**
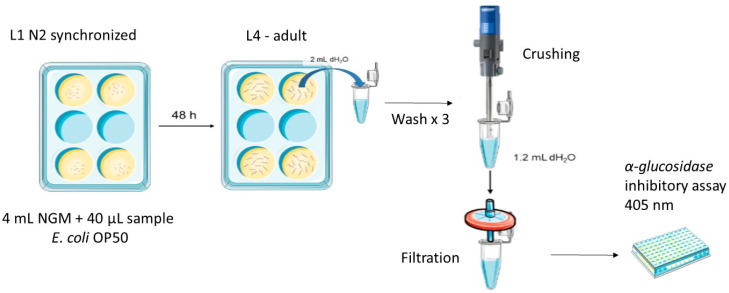
Scheme of the method adapted for the quantification of hypoglycemic activity in vivo.

Next, 100 µL of *E. coli* OP50 was seeded into each well as the worm food source, and 2000 L1 synchronized worms were placed in each well. After 48 h, they were collected with 2 mL of sterile water per plate, washed three times and pelleted by centrifugation (314 g/4 min/20 °C). The worms were then resuspended in 1.75 mL of sterile water and crushed for 20 s at maximum power using Ultra-Turrax^®^ T25 (IKA-Werke GmbH & Co KG, Staufen, Germany). The final solution was filtered through a 0.45 µm filter (Figure 5). Finally, the in vivo activity assay was performed in the same manner as previously described for the quantification of in vitro activity.

### 3.5. Statistical Analysis

All experiments were performed in triplicate. Means, standard deviations and graphs were calculated using Microsoft Excel 2013 (Microsoft Corp., Redmond, WA, USA). Statistical analysis was performed using Stata v.12 (StataCorp LLC, College Station, TX, USA). Normality was checked using the Shapiro–Wilkinson test, and differences were estimated by ANOVA followed by a post hoc pairwise comparison test using Tukey’s method (95% CL) or post-estimation margins to check for interactions between groups. Differences with *p* < 0.05 were considered statistically significant.

## 4. Conclusions

In this work, a novel, simple and reliable method was developed to directly determine antidiabetic activities in the in vivo model *C. elegans*. The adaptation of this approach to other activities would also allow for the attribution with certainty of functional and/or pharmacological effects derived from the ingestion of a compound that is not fluorimetrically traceable.

This approach has allowed for the analysis of two oral pharmaceutical forms with a hydroalcoholic extract of oregano, hard gelatin capsules and lyophilized powder for suspension in water. After simulating the intestinal digestion process, the results showed that the lyophilized powder shows better hypoglycemic activity. The results obtained with *O. vulgare* open up a new approach in research on antidiabetic compounds that could be administered orally.

## Data Availability

The original contributions presented in this study are included in the article; further inquiries can be directed to the corresponding author.
